# Clinical characteristics and camouflaging behaviors in children and adolescents with late-diagnosed autism spectrum disorder: a retrospective descriptive study

**DOI:** 10.3389/frcha.2026.1910194

**Published:** 2026-10-08

**Authors:** Nazlı Burcu Özbaran, Zeynep İrem Şener, Seda Sarı, Gülenay Korkmaz Şahin

**Affiliations:** 1 Department of Child and Adolescent Psychiatry, Ege University Faculty of Medicine, İzmir, Türkiye; 2 Department of Child and Adolescent Psychiatry, Dr. Behcet Uz Pedıatrıc Dıseases and Surgery Traınıng and Research Hospıtal, Izmir, Türkiye

**Keywords:** adolescents, autism spectrum disorder, camouflaging, child, delayed diagnosis, masking

## Abstract

**Purpose:**

Autism is a heterogeneous neurodevelopmental condition characterized by persistent difficulties in social communication and interaction, together with restricted and repetitive patterns of behavior, interests, or activities. Camouflaging-related behaviors and co-occurring psychiatric disorders may make autistic features less visible and contribute to delayed recognition. This study aimed to examine clinical characteristics and camouflaging-related behaviors among children and adolescents with late-diagnosed autism.

**Methods:**

This retrospective chart review included 46 children and adolescents aged 6–21 years who received an ASD diagnosis after the age of six. Autism diagnoses had been confirmed using a standardized observational assessment. Data were retrospectively extracted using structured case report forms based on clinical records, parental reports, and literature-informed camouflaging-related clinical features.

**Results:**

Attention-Deficit/Hyperactivity Disorder was the most common co-occurring psychiatric condition. Masking-related social adaptation behaviors were more common among girls. Giftedness and easy temperament were also observed more frequently in girls and were identified among factors associated with delayed recognition. Boys showed greater behavioral difficulties than girls. Clinician-rated camouflaging scores demonstrated a moderate positive correlation with post-diagnosis Global Assessment Scale scores (*r* = 0.38).

**Conclusions:**

The findings describe a clinically heterogeneous group of late-diagnosed children and adolescents and suggest that camouflaging-related behaviors, co-occurring psychiatric conditions, and contextual factors may reduce the visibility of autistic features. Given the retrospective design, small sample size, and use of a clinician-developed measure of camouflaging-related features, the findings should be interpreted as exploratory.

What is already known: Late diagnosis of autism spectrum disorder (ASD) remains common, particularly among females and individuals with less overt autistic presentations. Camouflaging has been proposed as a contributor to delayed recognition, although pediatric evidence remains limited.

What this study adds: This retrospective clinical study suggests that ADHD-related diagnostic overshadowing and camouflaging-related social adaptation behaviors may contribute to delayed ASD recognition in children and adolescents, with notable sex-related differences in presentation.

What is relevant: These findings may inform clinical practice by encouraging greater attention to less overt ASD presentations, particularly in youth presenting with psychiatric comorbidities. Further prospective research using developmentally appropriate pediatric measures is needed.

## Introduction

Autism Spectrum Disorder (ASD) is a neurodevelopmental disorder characterized by impairments in social communication and interaction, alongside restricted and repetitive behaviors (1). These features vary considerably in their presentation and severity and may be accompanied by differences in language, cognitive ability, adaptive functioning, sensory processing, emotional, behavioral, and neurodevelopmental challenges (2, 3).

Early diagnosis of autism improves coping mechanisms by providing access to appropriate support services, educational resources, and community programs, reducing parental stress, and enhancing adult independence (4). Missed diagnoses, however, restrict access to crucial early interventions and support during critical developmental periods (5). Early identification is therefore closely linked to effective developmental surveillance and screening. Autism-specific screening at 18 and 24 months, alongside ongoing developmental surveillance, has been recommended to facilitate the identification of children who may require comprehensive diagnostic assessment (6). Screening instruments do not themselves establish an autism diagnosis but can facilitate the early identification of autistic traits and subsequent referral for clinical evaluation (7). Moreover, behavioral markers associated with autism, including differences in joint attention, communication, gestures, and pretend play, may be detectable as early as the first year of life (8). Some studies reveal that a significant number of children do not receive a diagnosis until they are of school age (9). Higher symptom severity (10), higher socioeconomic status (11), and increased parental involvement (12) have been linked to early diagnosis.

Beyond access to intervention, receiving an autism diagnosis also initiates an adjustment process for the family. An earlier diagnosis may provide parents with an earlier opportunity to understand their child's developmental profile and begin processing the diagnosis. Parental resolution refers to the process through which parents integrate the diagnosis and develop a more balanced understanding of their child's strengths and difficulties. Resolution of the diagnosis has been associated with more adaptive parental representations, lower parenting stress, closer parent–child relationships, and more secure attachment patterns (13, 14). However, resolution is a complex process and should not be assumed to occur solely as a function of the time elapsed since diagnosis.

In recent years, diagnoses of autism spectrum disorder (ASD) during adulthood have been increasingly reported, particularly among females and individuals with high cognitive functioning (15). Late ASD diagnosis in children has been attributed to several factors, including children with better-developed cognitive abilities, families from more impoverished backgrounds (16) and milder autistic features (17, 18). However, there is currently no consensus on what constitutes a “late” autism diagnosis, and the age thresholds used across studies vary considerably depending on the population and research context (19). This variability may partly reflect differences in developmental stage, access to early intervention, and transitions between preschool and school-based services. Notably, age six has also been used as a meaningful threshold in previous research because it broadly corresponds to the transition from early intervention to school-age services (19, 20).

### Comorbidities and diagnostic overshadowing in autism spectrum disorder

Comorbid psychiatric disorders play a major role in the delayed diagnosis of autism spectrum disorder (ASD), by creating diagnostic overshadowing. Attention Deficit Hyperactivity Disorder (ADHD) has repeatedly been identified as the most common primary diagnosis recorded before an eventual ASD diagnosis (21). Kentrou et al. (22) reported that the delay in ASD diagnosis was longer in females with pre-existing ADHD compared to males, suggesting that overlapping symptoms between ADHD and ASD, or the predominance of ADHD symptoms, may obscure core autistic features and delay its identification.

### Camouflaging

Camouflaging refers to the use of coping strategies in social situations to conceal behaviors associated with ASD, including overt techniques to appear socially competent and prevent others from noticing social challenges (15).

Camouflaging strategies can facilitate social acceptance but come at significant psychological costs. Prolonged engagement in camouflaging is associated with increased anxiety, depression, and burnout, particularly among females, who tend to report higher camouflaging scores than males (23, 24). Camouflaging may also delay ASD diagnosis until social demands exceed coping capacity, leading to crisis presentations that trigger psychiatric evaluation (24). This sustained effort to maintain a neurotypical façade can reduce opportunities for timely support and exacerbate mental health difficulties over time (25, 26). However, debates remain regarding the validity of the camouflaging construct and whether it should be conceptualized as masking of symptoms or simply the absence of clinically significant traits (24). Overall, the literature underscores that while camouflaging may temporarily reduce the visibility of autistic traits, it carries significant long-term risks for psychological well-being.

Camouflaging is thought to be more common among women, potentially contributing to delayed diagnosis or misdiagnoses (27). In terms of strategies, autistic girls are more likely to engage in socially oriented camouflaging behaviors, such as imitating peers’ gestures and facial expressions, memorizing conversational scripts, and closely observing social interactions to learn norms (28).

In contrast, autistic boys are more likely to employ camouflaging through the suppression of atypical behaviors (e.g., stimming) and by limiting social engagement to reduce the visibility of their autistic traits, rather than through active imitation (28).

Taken together, the literature suggests that delayed autism diagnosis in children and adolescents may reflect the interplay of several domains, including camouflaging behaviors, individual and contextual characteristics, and co-occurring psychiatric conditions. Accordingly, examining these domains together may provide a more comprehensive clinical characterization of children and adolescents who receive an ASD diagnosis later in development. To date, research on the impact of camouflaging on diagnostic processes has been limited, with most studies focusing on adult populations. Therefore, the present study aimed to examine camouflaging behaviors in children and adolescents with delayed ASD diagnosis. For this purpose, clinical records were retrospectively reviewed. Within this framework, it was hypothesized that (i) camouflaging behaviors would be more prevalent among females, (ii) individual and contextual factors, including cognitive characteristics, temperament, family-related factors, social adaptation, and delayed recognition by families, schools, or healthcare professionals, would be associated with delays in the diagnostic process, and (iii) camouflaging behaviors and diagnostic delay would be associated with co-occurring psychiatric conditions, particularly ADHD, anxiety disorders, OCD, and MDD.

## Material and methods

### Procedure

This study was designed as a retrospective observational chart review conducted in a child and adolescent psychiatry clinic with established expertise in ASD monitoring and treatment. A total of 46 children and adolescents aged 6 to 21 years were included to investigate camouflaging and masking behaviors, as well as compensatory strategies, in the context of late ASD diagnosis. The data were obtained from past medical records; therefore, the current ages of some participants exceeded 18 years at the time of review. All participants had received an ASD diagnosis after the age of 6 at the Department of Child and Adolescent Psychiatry, Ege University.

The inclusion criteria were an ASD diagnosis at or after the age of six since 2018, follow-up by a clinician and faculty member experienced in autism, and voluntary agreement to participate. Data were obtained retrospectively through a review of medical records between April and September 2024. Throughout the manuscript, the term “current” refers to the participant's status at the time of data collection, based on the most recent clinical information available during the study period.

The diagnosis of ASD was established according to the Diagnostic and Statistical Manual of Mental Disorders, Fifth Edition (DSM-5). ASD diagnoses were verified using the Autism Diagnostic Observation Schedule, Second Edition (ADOS-2)-based assessment, and comorbid psychiatric disorders were assessed using the Schedule for Affective Disorders and Schizophrenia for School-Age Children Present and Lifetime Version—Turkish Adaptation (K-SADS). Autism severity was evaluated using the Childhood Autism Rating Scale (CARS). Overall functioning and illness severity were assessed using the Global Assessment Scale (GAS) and the Clinical Global Impression (CGI), respectively. In addition, WISC-R and WISC-IV results available in the clinical records were documented.

Camouflaging, masking, compensation, and reasons for delayed diagnosis were examined using a Case Report Form (CRF) developed based on a review of the literature on camouflaging in autism, our clinical experience with children and adolescents with ASD, and family reports documented in the clinical records (29).

The CRF is a detailed sociodemographic data form designed to explore camouflaging, masking, and compensation characteristics, as well as reasons for delayed diagnosis, through questions directed to the family and the clinician following the cases. Camouflaging, masking, and compensation characteristics were interpreted using a Visual Analog Scale (VAS) ranging from 0 to 10 based on family reports and clinicians' notes documented in the clinical records (30).

In addition, the reasons for delayed autism diagnosis were categorized and presented to the families (Supplementary Appendix S1).

### Ethical considerations

The study adhered to the principles outlined in the Helsinki Declaration. Approval for this study was obtained from the Ege University Medical Research Ethics Committee with the approval number 24−10.2T/28. Voluntary informed consent was obtained from all participants and their families.

### Data collection tools

Kiddie Schedule for Affective Disorders and Schizophrenia for School-Age Children—Present and Lifetime Version (K-SADS-PL): This semi-structured interview guide has been updated according to the DSM-V diagnostic criteria by Kaufman and colleagues (31). Its Turkish adaptation was made by Ünal and colleagues (32).

Clinical Global Impressions Scale (CGI): This scale evaluates the severity of the disease, how well the patient responds to treatment, treatment adherence, and improvement in disease symptoms. The clinician rates the severity of the illness and improvement in symptoms on a Likert scale ranging from 1 to 7. High scores indicate worse functionality (33). In this study, CGI score records before and after the diagnosis of ASD were evaluated.

Global Assessment Scale (GAS): This scale is used to assess functionality in both patient and healthy populations (34). The obtained scores range from 0 to 100, with higher scores indicating better functionality. GAS results were categorized as follows by Köhler and colleagues: scores from 61 to 100 indicate good functionality, scores from 31 to 60 indicate moderate functionality, and scores below 30 indicate poor functionality (35). In the study, GAS values in patient records before and after the diagnosis of ASD were evaluated.

Childhood Autism Rating Scale (CARS): The Childhood Autism Rating Scale, developed in 1971, is widely used in diagnosing autism and distinguishing other developmental disorders. The scale consists of 15 items, each scored on a half-point scale from 1 to 4. The total score ranges from 15 to 60. Children with a score over 30 receive an autism diagnosis, and as the score increases, the severity of the disorder also increases. Scores between 30−36 indicate mild to moderate autism, and scores over 37 indicate severe autism (36). This scale was applied to all cases in the study.

Autism Diagnostic Observation Schedule, Second Edition (ADOS-2): ADOS-2 is a semi-structured, standardized observational tool designed to assess ASD across all age groups and support diagnosis. The program consists of five developmentally-based modules, each containing a different combination of activities based on developmental age and expressive language skills (37). Each case was evaluated for ASD diagnosis using an ADOS-based clinical assessment conducted by a senior child and adolescent psychiatrist with more than 20 years of expertise in autism spectrum disorder.

Case Report Form (CRF): This form was developed based on information obtained from clinical records regarding camouflaging, masking, compensation, and reasons for delayed autism diagnosis, together with a review of the relevant literature in this field (29). The CRF is a comprehensive sociodemographic and clinical data collection tool addressing these domains through parallel questions posed to both families and the clinicians following the cases. The form includes sections on sociodemographic and clinical variables, chronic medical conditions, psychiatric diagnoses, age at ASD diagnosis, previous psychiatric diagnoses, psychiatric follow-up history, psychotropic medication use, details of the first psychiatric referral, families' statements regarding the reasons for late diagnosis of ASD and clinician's impressions from previous evaluations. Camouflaging behaviors are evaluated in terms of their presence, levels, compensatory strategies, masking behavior. Extended case characteristics include the parent–clinician relationship, treatment adherence, problem areas and their severity at the time of data collection, educational background, therapy history, early developmental behaviors and neurodevelopmental history. In the form, camouflaging level, masking level, and the extent of use of compensatory strategies were scored using VAS. (Supplementary Appendix S1.)

Visual Analog Scale (VAS): A 0–10 scale used to quantify subjective experiences (38). In this study, the VAS was used to assess camouflaging, masking, and compensation characteristics and to evaluate the level of concern reported by families regarding problem areas identified from clinical records.

### Statistical analysis

Descriptive statistics were presented as mean, standard deviation, median, minimum, maximum, frequency, and percentage values. The normality assumption for quantitative variables was evaluated using the Shapiro–Wilk test. Independent samples *t*-tests were used for normally distributed variables, whereas Mann–Whitney *U*-tests were applied for variables that did not meet normality assumptions. Correlations between quantitative variables were assessed using Spearman's rho correlation coefficient. Statistical analyses were performed using IBM SPSS Statistics 25.0 (IBM SPSS Statistics for Windows, Version 25.0. Armonk, NY: IBM Corp.). Partial correlation analysis was performed to examine the association between camouflaging rate and follow-up GAS score while controlling for baseline GAS. A multiple linear regression analysis was conducted with follow-up CGI score as the dependent variable and camouflaging rate, CARS score, age at ASD diagnosis, and baseline CGI score as independent variables. A significance level of *p* < .05 was adopted for all analyses.

## Results

### Sociodemographic findings and educational status

Data from 46 cases meeting the inclusion criteria are presented in this study. Sociodemographic data of the cases are shown in Table 1. Of the cases, 23.90% were female, and 76.10% were male. The mean age of the cases was 13.68 ± 3.73. Regarding the educational status of the cases, 3 cases (6.50%) were in preschool, 9 cases (19.60%) were in primary school, 11 cases (23.90%) were in middle school, and 23 cases (50%) were in high school. Following the late ASD diagnosis, all cases were referred for special education for their social skills development. At the time of data collection, 65.20% (30 cases) continued to receive special education, while 26.20% (12 cases) had never received special education and 8.70% (4 cases) had previously attended but no longer continued. The content of special education included social skills training for 52.20% (24 cases), social skills and cognitive support training for 15.20% (7 cases), social skills and cognitive behavioral therapy for 2.20% (1 case), cognitive support training for 2.20% (1 case), and specific learning disability support training for 4.30% (2 cases). Furthermore, 34.80% (16 cases) were currently receiving sensory integration therapy, while 6.50% (3 cases) had previously received sensory integration therapy.

**Table 1:** Sociodemographic characteristics of the cases.

Characteristic	*N* (%)			
Family Type				
Nuclear	33 (71.70)			
Extended	1 (2.20)			
Broken	12 (26.10)			
Place of Residence				
City center	38 (82.60)			
District	8 (17.40)			
Income Level				
Below minimum wage	1 (2.20)			
Minimum wage	1 (2.20)			
Low-middle	16 (34.80)			
Upper-middle	21 (45.70)			
High	7 (15.20)			
Gender				
Female	11 (23.90)			
Male	35 (76.10)			

	Mean ± SD	Min-Max	Test Statistic	p*
Age				
Female	14.45 ± 3.88	9–21	0.78	0.44*
Male	13.44 ± 3.70	6.5–20		
Total	13.68 ± 3.73	6.5–21		

*Independent Two-Sample *T*-Test. *Socioeconomic status was determined based on families reported income levels.

### Clinical characteristics

Data regarding the clinical status of the cases are presented in Table 2. ADHD was the most commonly observed comorbidity, followed by anxiety disorders, obsessive compulsive disorder and major depressive disorder. Additionally, 3 cases (6.5%) had gender dysphoria, 1 case (2.2%) had a speech disorder, 5 cases (10.9%) had tic disorders, and 1 case (2.2%) had an eating disorder (Table 2).

**Table 2:** Clinical characteristics of the cases.

Characteristic	*N* (%)
Presence of chronic illness	9 (19.60)
Presence of psychiatric disorder	
ASD	46 (100)
ADHD	41 (89.10)
Anxiety disorder	12 (26.10)
MDD	8 (17.40)
SLD	7 (15.20)
OCD	8 (17.40)
Use of psychotropics	40 (87)
Types of psychotropics used	
MPH	9 (19.56)
AP	33 (71.73)
ATX	9 (19.56)
Guanfacine	7 (15.21)
SSRI	21 (45.65)
MS	1 (2.17)

ASD, Autism Spectrum Disorder; ADHD, Attention Deficit and Hyperactivity Disorder; SLD, Specific Learning Disorder; MDD, Major Depressive Disorder; OCD, Obsessive Compulsive Disorder; MPH, Methylphenidate; AP, Antipsychotic; ATX, Atomoxetine; SSRI, Selective Serotonin Reuptake Inhibitor; MS, Mood Stabilizer.

None of the cases was diagnosed with psychotic disorder. Of the cases, 87% (40 cases) were using psychotropic medications. In terms of cognitive function, 13.04% (6 cases) had superior intellectual capacity, 15.21% (7 cases) had high intellectual capacity, 2.17% (1 case) had borderline intellectual capacity, 4.30% (2 cases) had mild intellectual disability, and 65.21% (30 cases) had normal intellectual capacity. Additionally, 19.60% (9 cases) had chronic medical conditions, including asthma, hypothyroidism, gastritis, hypophosphatemia, lactose intolerance, 46 XYY chromosomal anomaly, and osteoporosis.

### Psychiatric follow-up history

All participants in this study received their diagnosis of Autism Spectrum Disorder (ASD) from a child and adolescent psychiatrist with over 20 years of experience in the field. Data regarding the psychiatric history and follow-up of the cases are presented in Table 3. The mean age at which the cases received an ASD diagnosis was 11.68 ± 3.55 years. There was no significant difference in the age of diagnosis between genders. Only three participants (6.5%) sought psychiatric help on their own initiative. For the remaining participants, the initial psychiatric referral was initiated by the family (*n* = 21, 45.6%), a teacher (*n* = 12, 26.0%), a psychologist (*n* = 6, 13.0%), or a pediatrician (*n* = 4, 8.7%).

**Table 3:** Psychiatric follow-up history of the cases.

Variable	Mean ± SD	Min-Max	*N* (%)	Test Statistic	*p* ^a^
Age at first referral to psychiatry	8.12 ± 3.55	2–14			
Years of experience of the first psychiatrist seen	14.14 ± 7.39	1–20			
Age at ASD diagnosis				1.39	0.18^a^
Female	12.91 ± 3.27	8–18			
Male	11.30 ± 3.59	6–18			
Total	11.68 ± 3.55	6–18			
Duration in psychiatric follow-up (months)				−2.22	0.26^b^
Female	27.45 ± 23.42	1–72			
Male	63 ± 48,40	1.5–156			
Total	54.58 ± 46.13	1–156			
Origin of initial psychiatric referral					
Teacher			12 (26)		
Pediatrician			4 (8.70)		
Family			21 (45.60)		
Case him/herself			3 (6.50)		
Psychologist			6 (13)		
Reasons for delay in ASD diagnosis					
Camouflage			36 (78.30)		
Not diagnosed by physician			5 (10.90)		
Family denial of diagnosis			4 (8.70)		
Psychologist not referring			1 (2.20)		
Family suspicion of ASD before age 6					
Present			18 (39.10)		
Absent			28 (60.90)		
Families’ statements regarding the reasons for late diagnosis of ASD (with family suspicion before age 6)					
Normal			1 (7.14)		
Mild ASD risk			3 (21.42)		
Non-ASD diagnosis			9 (64.28)		
Lack of social communication skills			1 (7.14)		
Diagnoses before receiving an ASD diagnosis					
ADHD			22 (47.80)		
Non-diagnosed			9 (19.60)		
Anxiety disorder			4 (8.70)		
OCD			2 (4.30)		
MDD			1 (2.20)		
Eating disorder (*5 cases were diagnosed with ASD at their first visit*)			1(2.20)		

a Independent samples *t*-test.

b Mann–Whitney *U*-test.

ASD, Autism Spectrum Disorder; MDD, Major Depressive Disorder; OCD, Obsessive Compulsive.

### Diagnostic overshadowing and masking characteristics

The distribution of masking characteristics by gender is shown in Graph 1. Social adaptation behavior was the only masking-related characteristic that differed significantly between girls and boys, and it was more frequent in girls than in boys (*p* = 0.001, *t* = −3.42).

### Compensatory factors

Compensatory factors were categorized as high intelligence, giftedness, older age, the presence of supportive friends, maternal support, environmental support, family support, and an easy temperament based on information obtained from clinical records. These are shown in Graph 2. Giftedness and easy temperament were found to be compensatory strategies with differences between girls and boys, with girls showing more of these traits compared to boys (*p* = 0.02* *t* = −3.08) (*p* = 0.04* *t* = −2.04). No gender differences were found for high intelligence, older age, supportive friends, maternal support, environmental support, or family support.

### Families' statements regarding the reasons for late diagnosis of ASD

When families were asked about the reasons for the delayed diagnosis, the following reasons were reported: 11 cases (23.90%) for female gender, 20 cases (43.50%) for high intelligence, 18 cases (39.10%) for late referral to a psychiatrist, 13 cases (28.30%) for easy temperament, 12 cases (26.10%) for teachers lacking sufficient knowledge about ASD, 10 cases (21.70%) for good academic performance, 14 cases (30.40%) for having at least one individual with similar traits in the family, 11 cases (23.90%) due to the child's social adaptation behavior, 16 cases (34.80%) for the family's difficulty in accepting the ASD diagnosis, 6 cases (13%) for the child's superior abilities in some areas and 27 cases (58.70%) for not receiving an ASD diagnosis from consulted physicians. Among the reasons for diagnostic delay according to families, social adaptation behavior, giftedness, and easy temperament showed differences between genders, with boys experiencing more delays than girls (*p* < 0.001 *t* = −3.50), (*p* < 0.001 *t* = −3.62), (*p* = 0.02 *t* = −2.20).

### Problem areas of the cases

When the severity of problems in the social functioning, behavioral, academic, and obsessional domains was evaluated using the VAS based on family and clinician reports, the severity of social functioning problems was found to be 4.85 ± 2.82, behavioral problems 3.83 ± 3.27, academic problems 2.50 ± 3.15, and obsessional problems 4.65 ± 3.19. When comparing the severity of behavioral problems between girls and boys, boys showed significantly higher levels of behavioral problems (*p* = 0.01), while no significant differences were found in the other domains.

### Sensory sensitivity

Sensory sensitivity was present in 36 cases (78.30%), with 10 cases (21.70%) having visual sensitivity, 28 cases (60.90%) having auditory sensitivity, 20 cases (43.50%) having olfactory sensitivity, 18 cases (39.10%) having taste sensitivity, and 26 cases (56.50%) having tactile sensitivity.

### Non-suicidal self-injury (NSSI) and suicide attempts

Nine cases (19.56%) had a history of NSSI, with the most common method being self-cutting. Two cases (4.30%) had a history of suicide attempts, which involved jumping from heights and ingesting multiple drugs. Additionally, 8 cases (17.30%) reported passive suicidal thoughts.

### Social features of the cases

Regarding the past and current romantic relationship histories, 5 cases (10.86%) had a history of romantic relationships, and 3 cases (6.50%) were currently in a romantic relationship at the time of data collection. Thirty-three cases (71.70%) had a history of bullying, with 29 cases bullied by peers, 3 cases by both peers and teachers, and 1 case bullied only by teachers. Four cases (8.69%) had a history of bullying others.

### Differences in CGI/GAS scores before and after diagnosis in routine visit records (retrospective)

According to the data records, the initiation of appropriate treatment and interventions following the “late” diagnosis of ASD was associated with a decrease in CGI severity scores from 4.45 to 2.80 and an increase in GAS functioning scores from 52.71 to 73.04 (*p* < 0.001) (Table 4).

**Table 4:** Changes in CGI and GAS scores following late ASD diagnosis and initiation of appropriate interventions.

Measure	Pre	Post	Test Statistic	*p* ^a^
CGI	4.45	2.80	−5.88	<0.001
GAS	52.71	73.04	−5.83	<0.001

a Paired Samples t test.

CGI, Clinic Global Impression; GAS, Global Assessment Scale.

### Association between camouflaging behaviors and functional outcomes

Based on retrospective clinical records following the “late” diagnosis of ASD and the initiation of appropriate interventions and treatments, a moderate positive correlation was observed between the rate of camouflaging and the follow-up GAS score (rₛ = 0.38, *p* = 0.009). However, this association was no longer statistically significant after controlling for baseline GAS using partial correlation. The rate of camouflaging was also correlated with follow-up CGI score (rₛ = −0.36, *p* = 0.013), CARS score (rₛ = 0.43, *p* = 0.003), and baseline CGI score (rₛ = 0.33, *p* = 0.027).

In the linear regression model, with follow-up CGI score as the dependent variable and camouflaging rate, CARS score, age at ASD diagnosis, and baseline CGI score as independent variables, the overall model was statistically significant (*p* < 0.001, R = 0.694, adjusted R^2^ = 0.432). Camouflaging rate (B = −0.154, *p* = 0.034), CARS score (B = 0.049, *p* < 0.001), and age at ASD diagnosis (B = 0.049, *p* = 0.037) were significantly associated with follow-up CGI scores, whereas baseline CGI score was not significantly associated with the outcome.

## Discussion

In this study, we aimed to identify psychiatric, social, and individual factors associated with camouflaging, masking, and compensation behaviors in children and adolescents with late- diagnosed ASD. We examined the associations of social support factors with camouflaging and compensation, which have been scarcely addressed in the literature.

Few studies have explored the reasons and motivations for camouflaging in children and adolescents (39). It has been reported that autistic individuals generally use camouflaging as a tool to better adapt to neurotypical communities (15, 40). Therefore, understanding the types of camouflaging, the triggering factors, and the goals of camouflaging is crucial.

The mean age of the cases evaluated in our study was 13 years, with a mean age at ASD diagnosis of 11.68 ± 3.55 years. Studies have indicated the presence of camouflaging during adolescence (41, 42) or even earlier, in school-age children (43, 44). The age range in our study shows that camouflaging begins in school age. Recent studies report that the global average age for an ASD diagnosis is 5 years, with the age of diagnosis varying from 2.6 to 19.5 years (45). In the literature, the issue of delayed diagnosis in ASD is more pronounced in adult women; however, in our study, no significant difference was found between the diagnosis ages of girls and boys (46). This result, which contrasts with previous findings in the literature, may be related to the younger age of our sample compared to that of studies which have reported such a difference. However, this finding should also be interpreted cautiously given the gender imbalance in our sample (23.9% female and 76.1% male), which may have limited our ability to detect gender-related differences in age at diagnosis.

In our study, when evaluating the psychiatric disorders most frequently masking an ASD diagnosis, ADHD emerged as the most common, followed by anxiety disorders, obsessive-compulsive disorder (OCD), and major depressive disorder (MDD). Camouflaging has also been described in conditions such as ADHD and social anxiety disorder (47, 48). In our study, 89.1% of the cases had comorbid ADHD, a strikingly high rate consistent with previous reports (49). ADHD symptoms of hyperactivity and inattention may overshadow social withdrawal and communication difficulties, thereby contributing to diagnostic delays and missed diagnoses (50–52). In line with previous studies indicating that ADHD is the most frequent comorbidity in ASD and a potential contributor to diagnostic overshadowing (53, 54), our findings suggest that ADHD may play a crucial role as a contributing factor in delayed or missed ASD diagnosis.

Regarding the reasons for delayed diagnosis according to parental reports in our study, the most common reasons were the failure of healthcare providers to diagnose ASD (58.70%), high intelligence (43.50%), delayed consultation with psychiatrists (39.10%), difficulty accepting the diagnosis by families (34.80%), and having at least one family member with similar traits (30.40%). These findings are consistent with the results of the study conducted by Davidovitch et al. (55), in which delayed diagnoses were observed even when evaluations were carried out by a multidisciplinary team. These findings underscore the importance of post-graduate education focusing on the reasons for delayed diagnosis and the need for raising public awareness.

Camouflaging was examined in the study by Hull et al. (15), which evaluated camouflaging and compensation under the subsections of masking. In our study, these subsections were specifically analyzed for their associations with social acceptance in the child and adolescent group. Furthermore, maternal support was frequently identified in relation to camouflaging behavior in our sample, consistent with findings by Eisenberg et al. (56).

Mothers with adaptive emotional regulation skills are believed to not only model appropriate emotional regulation for their children but also create an environment in which children are more likely to express positive emotions.

Although the concept of social adaptation behavior has not been directly defined in the literature on ASD and masking, studies in psychiatric disorders such as depression and anxiety have reported that social adaptation behavior is influenced by gender differences and may lead to diagnostic overshadowing (57, 58). Inspired by this body of literature, in our study social adaptation behavior in ASD was defined as the child's attempts to imitate peers in order to communicate and engage in play, as well as their effort to participate in social settings when invited, even if they do not feel intrinsically inclined to do so. Notably, social adaptation behavior was the only camouflaging-related factor that differed significantly between girls and boys**,** with girls engaging in more effort than boys (*p* = 0.001, *t* = −3.42).

This constitutes one of the striking findings of our study. Previous research suggests that gender-based expectations and gender socialization during development may play a critical role in this difference. Social norms often encourage autistic girls to appear “more feminine” and “more social” than boys and to be more inclined to imitate or model socially appropriate behaviors according to gender norms (59). Such dynamics may increase the risk that core autistic features in girls remain hidden, thereby delaying or obscuring diagnosis. From a clinical perspective, our findings underscore the importance of carefully evaluating social adaptation strategies and compensatory behaviors in girls, rather than relying solely on overt symptom presentation. Future research should further explore how gendered socialization processes interact with individual masking strategies across development, and whether targeted assessment tools could capture these nuances to improve diagnostic equity.

In our study, 23.90% of the sample were females and 76.10% were males. Synthesizing the literature, it has been found that camouflaging is more common in females with autism, who are generally more aware of their social difficulties (60–63). Many studies have reported that camouflaging is a distinctive feature of autistic women (62, 64). While camouflaging strategies in males have been less studied, it has been reported that males tend to use different strategies, such as avoiding social communication or effectively managing physical space (28). The predominance of male cases in our sample provided valuable insights into the differences in camouflaging strategies between genders. Giftedness and easy temperament were the compensatory strategies found to differ significantly between girls and boys, with girls having more of these traits than boys (*p* = 0.02, *t* = −3.08) (*p* = 0.041, *t* = −2.04).

The severity of daily life problems related to social functioning, behavior and academic difficulties was assessed using the Visual Analog Scale (VAS) based on family and clinician reports, with social functioning issues being the most severe, followed by behavioral problems, academic difficulties, and obsessional problems. Our findings align with studies examining problem areas in autism (65–68). When comparing the severity of behavioral issues in girls and boys, males experienced more behavioral problems than females (*p* = 0.01). The finding that boys exhibited more behavioral problems than girls is particularly important, as it suggests that more overt and externalizing symptoms may lead to earlier recognition and diagnosis of autism in boys, whereas girls, who often display more subtle and internalized patterns of behavior, may be at greater risk for delayed diagnosis.

Sensory sensitivities were present in the majority of participants, including visual, auditory, olfactory, gustatory, and tactile sensitivities. The rate of sensory sensitivity in individuals with ASD varies between 45% and 95% in different studies (69–71). The sensory sensitivity rates observed in our study highlight the importance of assessing sensory sensitivities in the early diagnosis of ASD and may represent an important clinical clue for clinicians during the diagnostic process.

Nine cases had a history of Non-Suicidal Self-Injury (NSSI), with the most common method being self-injury. Two cases had a history of suicide attempts. Additionally, 17.30% (8 cases) reported passive suicidal thoughts. Previous studies have shown that autistic individuals are at markedly increased risk of self-injury and suicidality compared to the general population (72, 73). Importantly, such behaviors are often under-recognized, particularly in individuals with late or overshadowed diagnoses, where emotional and behavioral difficulties may mask core autistic features and delay appropriate intervention (52, 74). For cases with self-harm and suicide attempts, we suggest that ASD screening is critical for diagnosis and treatment.

Based on retrospective clinical records following the late diagnosis of ASD and the initiation of appropriate interventions and treatments, a positive correlation was observed between the rate of camouflaging and the follow-up GAS score (*r* = 0.38; *p* = 0.009). However, this association was no longer statistically significant after controlling for baseline GAS, suggesting that the observed relationship may partly reflect pre-existing differences in functioning. Camouflaging rate, CARS score, and age at ASD diagnosis were also significantly associated with post-treatment CGI scores. In contrast to adult studies linking camouflaging with increased psychological burden and autistic burnout (15, 23), our findings may reflect different developmental dynamics in children and adolescents. Given that late diagnosis has been associated with functional difficulties, such as reduced concentration in school due to sensory issues (75), these findings highlight the need for further longitudinal research to clarify the relationship between camouflaging and functional outcomes in children and adolescents with ASD.

This study examined psychiatric, social, and individual factors associated with camouflaging strategies in the pediatric population with autism spectrum disorder, with particular attention to social support mechanisms related to masking and compensation—areas that have been rarely explored in the existing literature. By addressing these dimensions, the study contributes to a more nuanced understanding of camouflaging behaviors and highlights their significance in children and adolescents with a late diagnosis. The changes observed in CGI and GAS scores following diagnosis and appropriate interventions suggest potential clinical benefits associated with accurate diagnosis and appropriate management.

### Strengths and limitations

This study has several strengths, including its focus on an understudied pediatric population with late-diagnosed ASD and the detailed clinical characterization of camouflaging-related behaviors and factors associated with diagnostic delay. In addition, the use of standardized diagnostic and clinical assessment tools within a specialized child and adolescent psychiatry setting strengthens the clinical characterization of the sample.

The study has several limitations that should be considered when interpreting the findings. First, the relatively small sample size (46 participants) limits the generalizability of the results, and the retrospective design does not allow prospective observation of changes in camouflaging behaviors over time. Second, the use of retrospective data poses challenges to data consistency, and psychiatric history obtained retrospectively may be subject to recall bias or inaccuracies in remembering past events. Third, conducting the study within a single cultural context raises questions about the applicability of the findings to more diverse populations. Finally, the lack of standardized instruments in the literature to assess camouflaging and masking behaviors in children and adolescents represents an important limitation. In this study, clinicians attempted to explore the clinical characteristics and reasons for delayed diagnosis using a clinician-developed Case Report Form (CRF), which may have introduced additional challenges. These limitations highlight the need for caution in interpreting the results and provide direction for future research.

## Data Availability

The original contributions presented in the study are included in the article/Supplementary Material, further inquiries can be directed to the corresponding author/s.
