# Supplementary material for: Clinical characteristics and camouflaging behaviors in children and adolescents with late-diagnosed autism spectrum disorder: a retrospective descriptive study
Source: Front Child Adolesc Psychiatry. 2026 Oct 8;5:1910194. doi: 10.3389/frcha.2026.1910194 (PMC13687046; doi:10.3389/frcha.2026.1910194)
Supplement: Supplementary file 1 [file Supplementaryfile1.docx]

**Case Report Form**

**Sociodemographic and Clinical Variables**

- Age
- Gender
- Presence of chronic medical conditions
- Current psychiatric diagnoses
- Age at ASD diagnosis
- Psychiatric diagnoses received before ASD was identified
- History of psychiatric follow-up
- History of psychotropic medication use
- Person who initiated first psychiatric referral
- Age at first psychiatric referral
- Years of experience of the first evaluating psychiatrist

**Additional Clinical and Diagnostic Variables**

- Presence of regression
- Families’ Statements Regarding the Reasons for Late Diagnosis of ASD *(Female gender, high intelligence, camouflaging, late referral to psychiatrist, easy temperament, teachers lacking sufficient knowledge about ASD, good academic performance, having at least one individual with similar traits in the family, family’s difficulty in accepting the ASD diagnosis, personal effort, giftedness, not receiving an ASD diagnosis from physicians)* *(Multiple options may be selected)*
- Presence of parental ASD suspicion before age 6
- Sought medical help after suspicion
- Clinician’s impressions from previous evaluations *(Considered normal, mild ASD risk, non-ASD diagnosis, lack of parental social stimulation)*

**Camouflaging**

- Presence of camouflaging
- Level of camouflaging *(Scored on a 0–10 scale using the Visual Analog Scale, VAS)*
- Camouflaging strategies used
- Level of camouflaging behavior *(Scored on VAS)*
- Level of compensation *(Scored on VAS)*

1) **Camouflaging Behavior** *(Multiple options may be selected)*

- Comorbid psychiatric conditions
- Physical health conditions
- Social adaptation behavior
- Family support
- Maternal support
- Good environmental adaption
- Younger age
- Presence of at least one individual with broad autism phenotype (BAP) in family
- Experience of living in a different cultural environment

2) **Compensatory Strategies** *(Multiple options may be selected)*

- High intelligence
- Family support
- Environmental support
- Easy temperament
- Giftedness
- Older age
- Supportive friends
- Maternal support
- Family support

**Extended Case Characteristics: Psychosocial, Educational, and Clinical Factors**

- Parent–Clinician relationship
- Current treatment adherence
- Current problem areas and severity *(e.g., Obsessions / Social / Behavioral / Academic) (Scored on VAS)*
- Sensory sensitivity
- History of romantic relationships
- History of being bullied
- History of Non-Suicidal Self-Injury (NSSI) and method
- History of Suicide Attempt and method
- Educational status
- History of special education / CBT / Occupational Therapy
- History of sleep, toilet training, and eating behaviors in ages 0–6

**Perinatal and Family Information**

- Family structure
- Socioeconomic status
- Number and birth order of siblings
- Use of assisted reproductive technologies (ART)
- History of physical or psychiatric ıllness and medication use during pregnancy
- Neurodevelopmental history

**Visual Analog Scale (VAS): A 0-10 scale used to quantify subjective experiences. In this study, the VAS was used to assess the level of camouflaging, masking, and compensation characteristics, as well as the severity of problem areas, in individuals diagnosed with ASD.*
